# Polymorphisms in the *VEGFA* and *VEGFR-2* genes and neovascular age-related macular degeneration

**Published:** 2009-12-10

**Authors:** Amy M. Fang, Aaron Y. Lee, Mukti Kulkarni, Melissa P. Osborn, Milam A. Brantley Jr.

**Affiliations:** 1 Department of Ophthalmology and Visual Sciences Washington University School of Medicine, St. Louis, MO; 2 Barnes Retina Institute, St. Louis, MO

## Abstract

**Purpose:**

Genetic factors influence an individual’s risk for developing neovascular age-related macular degeneration (AMD), a leading cause of irreversible blindness. Previous studies on the potential genetic link between AMD and vascular endothelial growth factor (VEGF), a key regulator of angiogenesis and vascular permeability, have yielded conflicting results. In the present case-control association study, we aimed to determine whether VEGF or its main receptor tyrosine kinase VEGFR-2 is genetically associated with neovascular AMD.

**Methods:**

A total of 515 Caucasian patients with neovascular AMD and 253 ethically-matched controls were genotyped for polymorphisms in the *VEGFA* and *VEGFR-2* genes. A tagging single nucleotide polymorphism (tSNP) approach was employed to cover each gene plus two kilobases on each side, spanning the promoter and 3′ untranslated regions. SNPs with a minimum allele frequency of 10% were covered by seven tSNPs in *VEGFA* and 20 tSNPs in *VEGFR-2*. Two *VEGFA* SNPs previously linked with AMD, rs1413711 and rs3025039, were also analyzed.

**Results:**

The 29 *VEGFA* and *VEGFR-2* SNPs analyzed in our cohort demonstrated no significant association with neovascular AMD. A single rare haplotype in the *VEGFR-2* gene was associated with the presence of neovascular AMD (p=0.034).

**Conclusions:**

This study is the first to investigate the association of *VEGFR-2* polymorphisms with AMD and evaluates *VEGFA* genetic variants in the largest neovascular AMD cohort to date. Despite the angiogenic and permeability-enhancing effects of VEGF/VEGFR-2 signaling, we found minimal evidence of a significant link between polymorphisms in the *VEGFA* and *VEGFR-2* genes and neovascular AMD.

## Introduction

Age-related macular degeneration (AMD) is a leading cause of irreversible blindness in individuals over the age of 50 in the Western world [1]. In addition to dietary and environmental risk factors, genetics influences AMD susceptibility [2]. Recently, single nucleotide polymorphisms (SNPs) in the complement factor H (*CFH*) gene [3-5] and the *ARMS2/HTRA1* locus [6,7] have been strongly linked to AMD. Common variants in the complement factor B (*CFB*)/complement component 2 (*C2*) [8,9] and complement component 3 (*C3*) [10-13] genes have also been associated with presence of AMD. Vascular endothelial growth factor (VEGF), coded for by the *VEGFA* gene, is a key mediator of angiogenesis and vascular permeability. Thus, VEGF is a logical candidate for genetically influencing AMD susceptibility, based on its functional relevance to AMD pathophysiology.

VEGF plays a key role in the angiogenesis, vascular leakage, and inflammation that is characteristic of the neovascular form of advanced AMD [14]. In recent years, the VEGF signaling pathway has been targeted for inhibition therapy to treat choroidal neovascularization secondary to AMD [15]. VEGF regulates angiogenesis in the vascular endothelium through the high-affinity receptor tyrosine kinases VEGFR-1 (Flt-1) and VEGFR-2 (Flk-1, KDR) [16]. Of these two receptors, VEGFR-2 is responsible for the majority of the angiogenic and permeability-enhancing effects of VEGF [17,18]. VEGFR-1 regulates VEGF activity in the vascular endothelium by preventing VEGF/VEGFR-2 binding [17].

A relationship between the *VEGFA* and *VEGFR-2* genes and neovascular AMD seems plausible, given the role of choroidal neovascularization in the pathophysiology of late-stage AMD and the importance of the receptor VEGFR-2 in the VEGF-signaling pathways that modulate angiogenesis and vascular permeability. Several small-scale case-control studies have reported associations between various SNPs in *VEGFA* and AMD [18-21], but these results have not been confirmed by subsequent studies [22-24]. While a recent analysis of *VEGFA* SNPs showed no associations with neovascular AMD, it did suggest that a three-SNP *VEGFA* haplotype increased the risk of neovascular AMD [25]. To our knowledge, the potential link between *VEGFR-2* and AMD has not been investigated. In this study, we focused on neovascular AMD, as its positive response to anti-VEGF therapy implicates the VEGF pathway in the pathogenesis of this AMD subtype. Using a tagging SNP (tSNP) approach in a large cohort of neovascular AMD patients, we aimed to determine if a genetic association exists between the *VEGFA* or *VEGFR-2* genes and neovascular AMD.

## Methods

### Subjects

This retrospective case-control association study was approved by the Washington University Human Research Protection Office and the Barnes Retina Institute Study Center. Research adhered to the tenets of the Declaration of Helsinki and was conducted in accordance with Health Insurance Portability and Accountability Act regulations. All participants were enrolled from the clinical offices of the Barnes Retina Institute, and informed consent was obtained before participation. Participants were identified through chart review, and only Caucasian patients with a diagnosis of neovascular AMD were included. Controls were identified as having no signs of AMD and were recruited from the same locations.

A total of 768 patients were genotyped for SNPs in the *VEGFA* and *VEGFR-2* genes using DNA extracted from mouthwash samples. Each participant provided buccal tissue samples by expectorating into 50 ml conical tubes (Falcon; BD Biosciences, San Jose, CA) after vigorously rinsing for 30 s with approximately 20 ml Scope mouthwash (Procter & Gamble, Cincinnati, OH). Genomic DNA was prepared from the collected buccal cells using the Gentra Puregene Buccal Cell Kit (Qiagen, Valencia, CA).

### Tagging single nucleotide polymorphism selection

We employed a tSNP approach to cover the *VEGFA* and *VEGFR-2* genes plus two kilobases (kb) on each side, including the promoter and 3′ untranslated regions. Using HapMap Project Build 36, we identified the SNPs in each gene with a minimum allele frequency (MAF) of 10% [18,22]. The minimum r^2^ value was set to 0.80, and genotypes for the Centre d’Etude du Polymorphisme Humain (CEPH) from Utah (CEU) population (Utah residents with ancestry from northern and western Europe) were used. We selected *VEGFA* tSNPs and *VEGFR-2* tSNPs covering the HapMap-identified SNPs through the Tagger algorithm. Using Sequenom MassARRAY technology (Sequenom Inc., San Diego, CA), study participants were genotyped for these tSNPs, as well as for two additional SNPs (rs1413711 and rs3025039) previously associated with AMD [19,20].

### Data analysis

Descriptive statistics for all demographic and clinical variables were calculated, and comparisons were made using the ANOVA test for means with continuous data (e.g., age) and the chi-square test for categorical data (e.g., gender). Genotyping data was loaded into Haploview in linkage format to generate allele frequencies, ratios, and p values based on a chi-square test for association of alleles [26]. The Hardy–Weinberg equilibrium test was used to confirm that genotypes fell within a standard distribution. Genotype distributions were analyzed by logistic regression, incorporating adjustments for age and gender. Haplotype blocks encompassing the tested SNPs were defined by 95% confidence intervals and were predicted using Haploview. Statistics were performed with SPSS (version 17; SPSS Inc., Chicago, IL). For all statistical analyses, p<0.05 was considered to be statistically significant, and multiple comparisons were adjusted for by the Bonferroni correction.

## Results

Using HapMap and Tagger for tSNP selection, we identified seven *VEGFA* tSNPs and 22 *VEGFR-2* tSNPs to cover both genes. Because two assays failed design, 20 tSNPs in *VEGFR-2* were selected for genotyping. The two failed tSNPs correlated poorly with other SNPs, and alternative SNPs could not be chosen. The 20 genotyped tSNPs in *VEGFR-2* had a mean r^2^ of 0.97 and captured 44 of the 46 SNPs with a MAF greater than 10%.

A total of 515 Caucasian patients with neovascular AMD and 253 ethnically-matched controls were genotyped for this study. Cases and controls had mean ages of 79.2 and 69.3 years, respectively (p<0.001). The case cohort consisted of a lower percentage of males (33.6%) than did the control cohort (43.9%; p=0.005). Adjustments for age and gender were incorporated in the genotype analysis of both *VEGFA* and *VEGFR-2*. All analyzed SNPs conformed to Hardy–Weinberg equilibrium in both the case and control populations. Allele distributions did not differ significantly between cases and controls for any of the nine *VEGFA* SNPs (Table 1). According to the genotype distribution for these SNPs (Table 2), one tSNP (rs699947) and one SNP previously associated with AMD (rs1413711) demonstrated significant uncorrected p values, but no significant association was found for any *VEGFA* SNP after correcting for multiple comparisons. Allele distributions for the 20 *VEGFR-2* tSNPs showed significant uncorrected p values for three tSNPs, but none remained significant after correcting for multiple comparisons (Table 3). No *VEGFR-2* tSNPs were associated with neovascular AMD by genotype distribution (Table 4).

**Table 1:** Allele distribution for single nucleotide polymorphisms in *VEGFA*

**SNP**	**Location in gene**	**Genomic location**	**Variation**	**Variant allele in controls, n (%)**	**Variant allele in cases, n (%)**	**Odds ratio (95% CI)**	**Unadjusted p-value**	**Adjusted p-value**
rs699947	5′ UTR	43844367	A to C	267 (52.8)	500 (48.5)	0.84 (0.68–1.05)	0.120	1.000
rs25648	Exon 1	43846955	C to T	98 (19.4)	188 (18.3)	0.93 (0.71–1.22)	0.598	1.000
rs1413711	Intron 1	43848656	G to A	240 (47.4)	528 (51.3)	1.17 (0.94–1.44)	0.158	1.000
rs833068	Intron 2	43850505	G to A	153 (30.2)	342 (33.2)	1.15 (0.91–1.44)	0.242	1.000
rs2146323	Intron 2	43853073	C to A	189 (37.4)	362 (35.1)	0.91 (0.73–1.13)	0.376	1.000
rs3025030	Intron 7	43858565	G to C	67 (13.2)	137 (13.3)	1.01 (0.73–1.38)	0.974	1.000
rs3025035	Intron 7	43859337	C to T	34 (6.7)	77 (7.5)	1.12 (0.74–1.70)	0.591	1.000
rs3025039	3′ UTR	43860514	C to T	66 (13.0)	137 (13.3)	1.02 (0.75–1.40)	0.889	1.000
rs10434	3′ UTR	43861190	G to A	246 (48.6)	484 (47.0)	0.94 (0.76–1.16)	0.549	1.000

A total of 515 patients with age-related macular degeneration (cases) and 253 ethnically-matched individuals without age-related macular degeneration (controls) were genotyped for nine single nucleotide polymorphisms (SNPs) in the *VEGFA* gene. The Location in gene column refers to the location of the SNP within the *VEGFA* gene. The Genomic location column identifies the specific location of each SNP on Chromosome 6. The Variation column indicates the nature of the polymorphism from the normal allele to the variant allele. The frequency of the minor allele for each SNP was compared in cases and controls by the chi-square test. Odds ratios, 95% confidence intervals (CI), and p-values are reported. Adjusted p-values have been corrected for multiple comparisons using the Bonferroni method.

**Table 2:** Genotype distribution for single nucleotide polymorphisms in *VEGFA*

**SNP**	**Genotype**	**OR**	**95% CI**		**Unadjusted p-value**	**Adjusted p-value**
rs699947	AC	1.02	0.66	1.57	0.025	0.223
	CC	1.81	1.08	3.05		
rs25648	CT	1.02	0.69	1.53	0.180	1.000
	TT	0.36	0.15	0.86		
rs1413711	GA	0.62	0.40	0.96	0.042	0.381
	AA	0.59	0.35	0.98		
rs833068	GA	1.38	0.95	2.02	0.237	1.000
	AA	1.17	0.65	2.15		
rs2146323	CA	0.82	0.56	1.20	0.326	1.000
	AA	0.82	0.48	1.40		
rs3025030	GC	1.10	0.72	1.70	0.254	1.000
	CC	2.88	0.76	14.69		
rs3025035	CT	1.19	0.69	2.10	0.230	1.000
	TT	1749	0.00	NA		
rs3025039	CT	1.12	0.74	1.72	0.296	1.000
	TT	2.48	0.62	13.08		
rs10434	GA	0.77	0.50	1.18	0.099	0.892
	AA	0.66	0.40	1.08		

Genotype analysis of *VEGFA* single nucleotide polymorphisms (SNPs) was performed by logistic regression, incorporating adjustments for age and gender. Odds ratios (OR), 95% confidence intervals (CI), and p-values are reported by genotype (normal/variant, variant/variant) for each tested SNP. Adjusted p-values have been corrected for multiple comparisons using the Bonferroni method.

**Table 3:** Allele distribution for tagging single nucleotide polymorphisms in *VEGFR-2*

**SNP**	**Location in gene**	**Genomic location**	**Variation**	**Variant allele in controls, n (%)**	**Variant allele in cases, n (%)**	**Odds ratio (95% CI)**	**Unadjusted p-value**	**Adjusted p-value**
rs7691507	3′ UTR	55637758	T to C	95 (18.8)	191 (18.5)	0.98 (0.75–1.29)	0.913	1.000
rs12642307	Intron 27	55646938	T to C	142 (28.1)	319 (31.0)	1.15 (0.91–1.45)	0.243	1.000
rs2125489	Intron 27	55648240	C to T	58 (11.5)	106 (10.3)	0.89 (0.63–1.24)	0.485	1.000
rs1531289	Intron 25	55649989	G to A	61 (28.9)	306 (29.7)	1.04 (0.82–1.32)	0.730	1.000
rs17709898	Intron 22	55652480	A to G	146 (39.1)	348 (33.8)	0.79 (0.64–0.99)	0.040	0.795
rs12505758	Intron 15	55661655	T to C	55 (10.9)	118 (11.5)	1.06 (0.76–1.49)	0.732	1.000
rs13109660	Intron 13	55665437	G to A	159 (31.4)	378 (36.7)	1.27 (1.01–1.59)	0.042	0.831
rs1870377	Exon 11	55667731	T to A	134 (26.5)	232 (22.5)	0.81 (0.63–1.03)	0.087	1.000
rs7654599	Intron 9	55670925	T to C	210 (41.5)	418 (40.6)	0.96 (0.78–1.20)	0.731	1.000
rs17085326	Intron 7	55672133	C to T	38 (7.5)	81 (7.9)	1.05 (0.70–1.57)	0.807	1.000
rs2034965	Intron 7	55672557	G to A	133 (26.3)	269 (26.1)	0.99 (0.78–1.26)	0.944	1.000
rs10020464	Intron 7	55673827	C to T	150 (29.6)	300 (29.1)	0.98 (0.77–1.23)	0.834	1.000
rs2305948	Exon 7	55674315	C to T	61 (12.1)	89 (8.6)	0.69 (0.49–0.97)	0.034	1.000
rs7692791	Intron 6	55674996	T to C	233 (46.1)	486 (47.2)	1.05 (0.85–1.30)	0.675	1.000
rs2305949	Exon 6	55675213	C to T	111 (21.9)	207 (20.1)	0.90 (0.69–1.16)	0.403	1.000
rs6837735	Intron 2	55680572	C to T	80 (15.8)	191 (18.5)	1.21 (0.91–1.61)	0.187	1.000
rs1531290	Intron 2	55681319	A to G	239 (47.2)	509 (49.4)	1.09 (0.88–1.35)	0.421	1.000
rs12502008	Intron 1	55685799	G to T	180 (35.6)	368 (35.7)	1.01 (0.81–1.26)	0.953	1.000
rs7667298	5′ UTR	55686488	T to C	226 (44.7)	486 (47.2)	1.11 (0.89–1.37)	0.352	1.000
rs2239702	5′ UTR	55686896	G to A	121 (24.9)	263 (25.5)	1.03 (0.81–1.32)	0.789	1.000

A total of 515 patients with age-related macular degeneration (cases) and 253 ethnically-matched individuals without age-related macular degeneration (controls) were genotyped for twenty single nucleotide polymorphisms (SNPs) in the *VEGFR-2* gene. The Location in gene column refers to the location of the SNP within the *VEGFR-2* gene. The Genomic location column identifies the specific location of each SNP on Chromosome 4. The Variation column indicates the nature of the polymorphism from the normal allele to the variant allele. The frequency of the minor allele for each SNP was compared in cases and controls by the chi-square test. Odds ratios, 95% confidence intervals (CI), and p-values are reported. Adjusted p-values have been corrected for multiple comparisons using the Bonferroni method.

**Table 4:** Genotype distribution for tagging single nucleotide polymorphisms in *VEGFR-2*

**SNP**	**Genotype**	**OR**	**95% CI**		**Unadjusted p-value**	**Adjusted p-value**
rs7691507	TC	1.04	0.71	1.55	0.602	1.000
	CC	1.37	0.53	3.91		
rs12642307	TC	1.11	0.77	1.62	0.306	1.000
	CC	1.40	0.74	2.76		
rs2125489	CT	0.83	0.54	1.29	0.482	1.000
	TT	1.23	0.19	24.17		
rs1531289	GA	1.11	0.76	1.63	0.360	1.000
	AA	1.34	0.70	2.63		
rs17709898	AG	0.77	0.52	1.13	0.068	1.000
	GG	0.63	0.37	1.09		
rs12505758	TC	0.91	0.57	1.44	0.548	1.000
	CC	5.68	0.99	107.98		
rs13109660	GA	1.09	0.75	1.58	0.271	1.000
	AA	1.47	0.80	2.78		
rs1870377	TA	0.96	0.66	1.42	0.527	1.000
	AA	0.76	0.37	1.56		
rs7654599	TC	0.72	0.48	1.08	0.672	1.000
	CC	1.00	0.58	1.76		
rs17085326	CT	0.85	0.51	1.43	0.665	1.000
	TT	1.21	0.23	9.16		
rs2034965	GA	1.13	0.77	1.65	0.506	1.000
	AA	1.18	0.55	2.63		
rs10020464	CT	1.24	0.85	1.81	0.516	1.000
	TT	1.02	0.55	1.93		
rs2305948	CT	0.85	0.53	1.39	0.250	1.000
	TT	0.43	0.10	1.80		
rs7692791	TC	1.23	0.80	1.88	0.834	1.000
	CC	0.94	0.58	1.53		
rs2305949	CT	1.05	0.71	1.57	0.605	1.000
	TT	0.62	0.27	1.46		
rs6837735	CT	1.22	0.82	1.82	0.209	1.000
	TT	1.65	0.57	5.47		
rs1531290	AG	0.90	0.58	1.37	0.605	1.000
	GG	1.15	0.70	1.91		
rs12502008	GT	0.86	0.59	1.26	0.449	1.000
	TT	0.85	0.47	1.58		
rs7667298	TC	0.90	0.57	1.41	0.259	1.000
	CC	0.76	0.46	1.24		
rs2239702	GA	1.15	0.79	1.66	0.465	1.000
	AA	1.17	0.57	2.52		

Genotype analysis of *VEGFR-2* tagging single nucleotide polymorphisms (tSNPs) was performed by logistic regression, incorporating adjustments for age and gender. Odds ratios (OR), 95% confidence intervals (CI), and p-values are reported by genotype (normal/variant, variant/variant) for each tested tSNP. Adjusted p-values have been corrected for multiple comparisons using the Bonferroni method.

**Figure 1:**
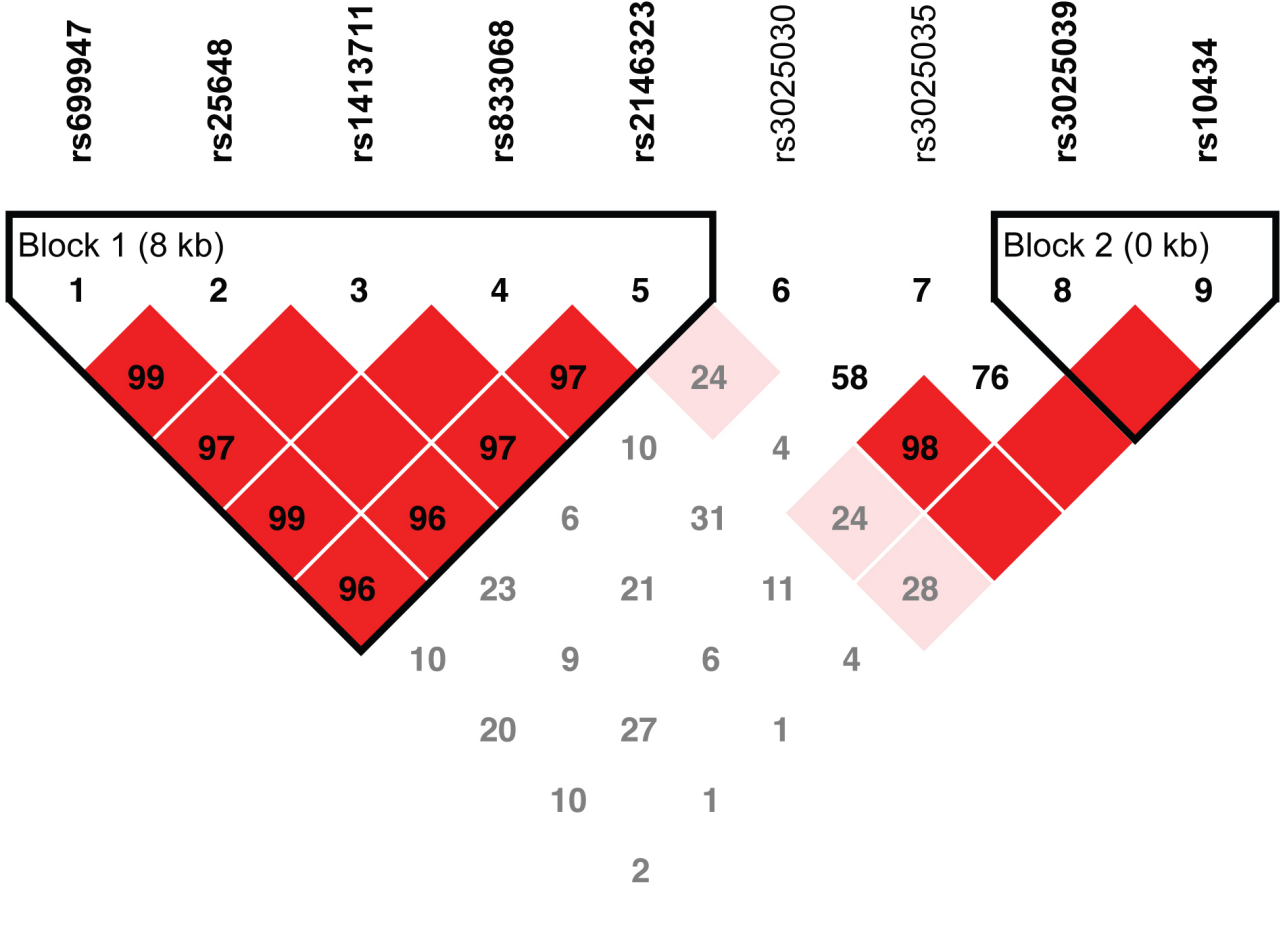
Linkage disequilibrium map of *VEGFA* single nucleotide polymorphisms. Two haplotype blocks (bolded) were identified for the nine single nucleotide polymorphisms (SNPs) in the vascular endothelial growth factor (*VEGFA*) gene. A linkage disequilibrium map of these haplotype blocks was generated using Haploview. Length of each block is provided in kilobases (kb), and pairwise linkage disequilibrium (D’) is given for each SNP combination. Dark red shading denotes D’ values greater than 0.80, and empty squares indicate D’ values of 1.0.

**Figure 2:**
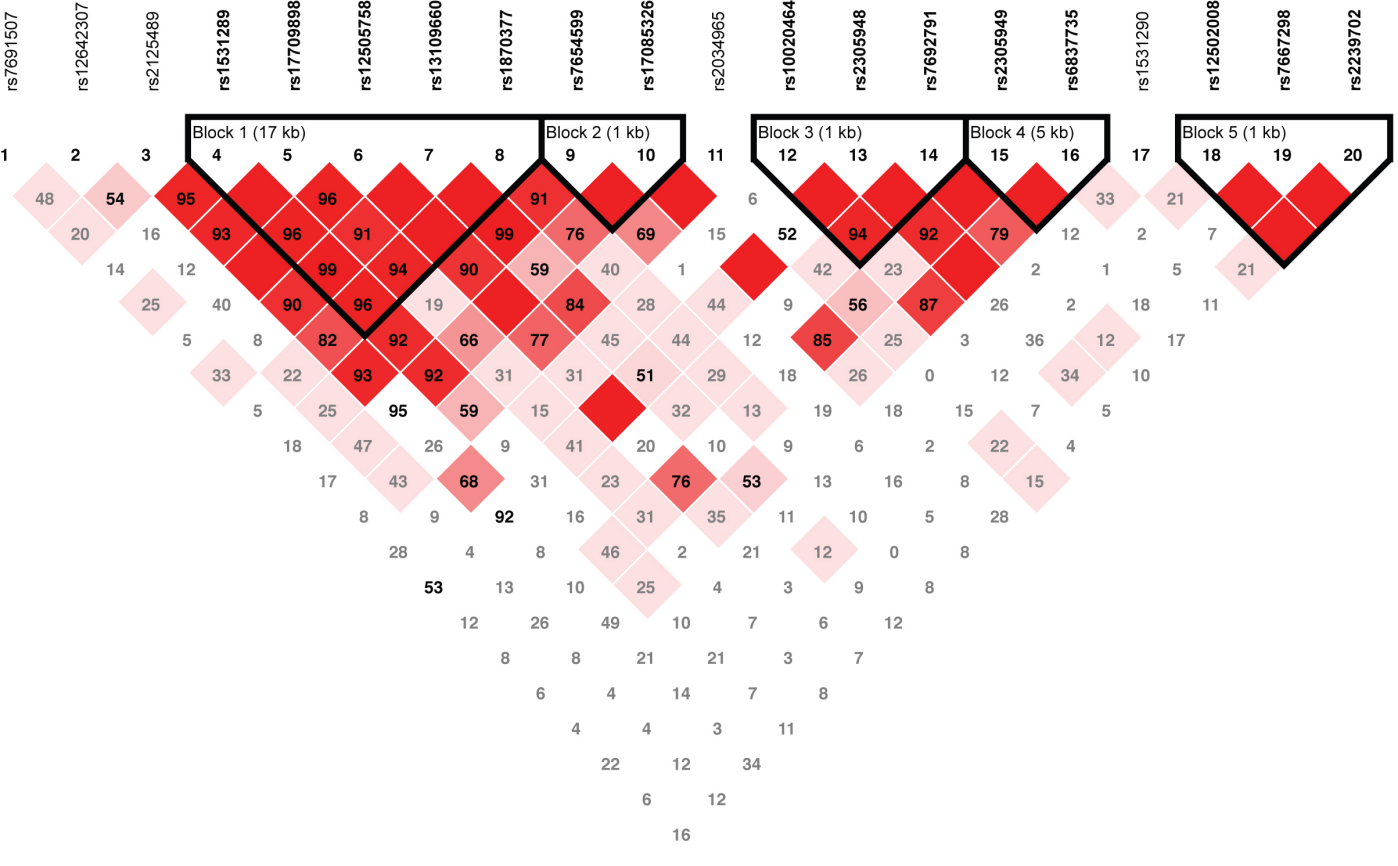
Linkage disequilibrium map of *VEGFR-2* tagging single nucleotide polymorphisms. Five haplotype blocks were identified for the 20 tagging single nucleotide polymorphisms (tSNPs) in the vascular endothelial growth factor receptor-2 (*VEGFR-2*) gene. A linkage disequilibrium map of these haplotype blocks was generated using Haploview. Length of each block is provided in kilobases (kb), and pairwise linkage disequilibrium (D’) is given for each SNP combination. Dark red shading denotes D’ values greater than 0.80, and empty squares indicate D’ values of 1.0.

Linkage disequilibrium-based haplotypes were defined for the *VEGFA* and *VEGFR-2* SNPs, resulting in two haplotype blocks for *VEGFA* (Figure 1) and five haplotype blocks for *VEGFR-2* (Figure 2). Haplotype analysis demonstrated no *VEGFA* haplotypes to be associated with neovascular AMD (Table 5) but did reveal a mild association of the rarest haplotype (TTT) in *VEGFR-2* haplotype block 3 with neovascular AMD (p=0.034, Table 6).

**Table 5:** Haplotype analysis of *VEGFA*

**SNP 1**	**SNP 2**	**SNP 3**	**SNP 4**	**SNP 5**	**Frequency in cases**	**Frequency in controls**	**p-value**
**Block 1**							
rs699947	rs25648	rs1413711	rs833068	rs2146323			
C	C	G	A	C	0.329	0.292	0.139
A	T	A	G	A	0.176	0.193	0.428
C	C	G	G	C	0.177	0.166	0.592
A	C	A	G	A	0.169	0.171	0.910
A	C	A	G	C	0.133	0.153	0.282
**Block 2**							
rs3025039	rs10434						
C	A				0.470	0.486	0.549
C	G				0.397	0.383	0.606
T	G				0.133	0.130	0.889

Two haplotype blocks were identified for the tested single nucleotide polymorphisms (SNPs) in *VEGFA* using Haploview. Haplotype frequencies in neovascular age-related macular degeneration patients (cases) and participants without age-related macular degeneration (controls) are reported with corresponding p-values. Haplotypes with frequencies above 0.01 are included in the table.

**Table 6:** Haplotype analysis of *VEGFR-2*

**SNP 1**	**SNP 2**	**SNP 3**	**SNP 4**	**SNP 5**	**Frequency in cases**	**Frequency in controls**	**p-value**
**Block 1**							
rs1531289	rs17709898	rs12505758	rs13109660	rs1870377			
G	A	T	A	T	0.351	0.304	0.066
G	G	T	G	A	0.217	0.257	0.087
A	A	T	G	T	0.182	0.182	0.992
G	G	T	G	T	0.105	0.123	0.302
A	A	C	G	T	0.113	0.102	0.514
G	G	T	A	T	0.015	0.008	0.248
**Block 2**							
rs7654599	rs17085326						
T	C				0.516	0.51	0.835
C	C				0.406	0.415	0.731
T	T				0.079	0.075	0.807
**Block 3**							
rs10020464	rs2305948	rs7692791					
C	C	C			0.463	0.455	0.772
C	C	T			0.246	0.248	0.910
T	C	T			0.196	0.170	0.230
T	T	T			0.086	0.121	0.034
**Block 4**							
rs2305949	rs6837735						
C	C				0.614	0.623	0.735
T	C				0.201	0.219	0.403
C	T				0.185	0.158	0.187
**Block 5**							
rs12502008	rs7667298	rs2239702					
T	C	G			0.357	0.356	0.953
G	T	A			0.255	0.249	0.789
G	T	G			0.217	0.198	0.394
G	C	G			0.171	0.198	0.199

Five haplotype blocks were identified for the tagging single nucleotide polymorphisms (tSNPs) in *VEGFR-2* using Haploview. Haplotype frequencies in neovascular age-related macular degeneration patients (cases) and participants without age-related macular degeneration (controls) are reported with corresponding p-values. Haplotypes with frequencies above 0.01 are included in the table.

## Discussion

We analyzed nine SNPs in *VEGFA* and 20 tSNPs in *VEGFR-2* using a tSNP approach designed to cover both genes. The results of this study show little evidence of association with neovascular AMD in our cohort of 515 Caucasian patients.

Previous investigation of *VEGFA* and AMD has yielded conflicting results. A few smaller-scale case-control studies have reported significant associations for various SNPs in *VEGFA* [18-21], but the validity of these results remains unconfirmed [22-24]. The only one of these studies to investigate a large neovascular AMD cohort (n=342) using a comprehensive tSNP approach found no link between *VEGFA* tSNPs and development of neovascular AMD [22]. More recently, a single haplotype was shown to be weakly associated with neovascular AMD in a moderately-sized cohort (n=211), although no associations were found with individual *VEGFA* SNPs [25]. In the present study, we found no association between neovascular AMD and *VEGFA* SNPs or haplotypes. These discrepant findings may be due to inadequate sample size in the early studies or to the ethnic composition of study populations. Results could also be biased by genotyping error or confounding effects due to statistically significant variables such as age, gender, or other SNPs known to be associated with AMD. As a whole, the studies performed to date provide little evidence that *VEGFA* polymorphisms exert any significant influence on risk of neovascular AMD.

This is the first study to investigate the relationship between variants in *VEGFR-2* and AMD. One of two receptor tyrosine kinases involved in VEGF signaling pathways, VEGFR-2 mediates the majority of the angiogenic and permeability-enhancing effects of VEGF [17]. The importance of VEGFR-2 in developmental angiogenesis and hematopoiesis, as demonstrated by the abnormal vasculature of *VEGFR-2* knockout mice [27], suggests a link between the VEGF/VEGFR-2 pathway and retinal pathology. While VEGF and its receptors play a key role in tumor angiogenesis and other pathological conditions [14,16,28,29], a limited number of gene association studies have been performed for VEGF receptor genes. Recently, polymorphisms in *VEGFR-1* and *VEGFR-2* were reported to be associated with sarcoidosis, an inflammatory condition with a hypothesized antigenic stimulus [30]. Variants in *VEGFR-2* have also been linked with heart disease and may influence the risk of developing breast cancer [31-33]. In our large neovascular AMD cohort, no associations were found for any *VEGFR-2* tSNPs by allele or genotype analysis. Haplotype analysis, however, did show a single rare haplotype to be mildly associated with AMD.

This study is limited by its retrospective design, which did not allow for assessment of the predictive value of *VEGFA* and *VEGFR-2*. However, in light of our negative findings, it is doubtful that a prospective study would contribute significantly to our knowledge base. This study was designed to investigate common polymorphisms that might be associated with neovascular AMD risk, and it remains possible that rare variants with MAFs less than 10% could play a role in neovascular AMD development. Due to tSNP assay failure, two of the 46 *VEGFR-2* SNPs with a MAF greater than 10% were poorly tagged. The 20 successful *VEGFR-2* tSNPs in our study covered the polymorphisms previously found to be associated with human diseases: rs1870377, rs2071559 (covered by rs7667298), rs2125489, rs2305948, rs7667298, and rs7691507 [30-33].

In summary, this study is the first to investigate the association of *VEGFR-2* polymorphisms with AMD and evaluates *VEGFA* genetic variants in the largest neovascular AMD cohort to date. Although both VEGF and its receptors have been implicated in the pathophysiology of diseases such as AMD, we found minimal evidence that polymorphisms in *VEGFA* and *VEGFR-2* contribute significantly to risk of neovascular AMD.
